# Brown-Séquard syndrome in a 11-year-old girl due to penetrating glass injury to the thoracic spine

**DOI:** 10.1007/s00590-012-1050-8

**Published:** 2012-07-19

**Authors:** M. Komarowska, W. Debek, J. A. Wojnar, A. Hermanowicz, M. Rogalski

**Affiliations:** 1 Department of Pediatric Surgery, Medical University of Bialystok, Bialystok, Poland; 2 Department of Pediatric Orthopaedics and Traumatology, Medical University of Bialystok, Bialystok, Poland

**Keywords:** Brown-Séquard syndrome, Spinal cord injury, Stab wound

## Abstract

Injuries in children are one of the most frequent causes of high morbidity and mortality, and they present a challenge to the treating physician. Fortunately, spinal trauma in pediatric patient is relatively rare. Brown-Séquard syndrome is a rare form of incomplete spinal cord injury consisting of ipsilateral upper motor neuron paralysis (hemiplegia) and loss of proprioception with contralateral pain and temperature sensation deficits resulting from hemisection or lateral injury to the spinal cord. A 11-year-old girl was admitted to our Pediatric Trauma Emergency Department after she had suffered a penetrating back injury. Neurological examination demonstrated left lower extremity paresis and moderate spastic paralysis of the right lower extremity. The examination showed loss of temperature sensation contralateral to and below the lesion. The examination of the pain sensation was difficult because the patient was in pain shock, but it was diminished on the side opposite to the damage. Multislice spiral computed tomography (MSCT) demonstrated a triangular foreign body in spinal canal at the level of the Th11–Th12. After a Th_11_–L_2_ laminectomy and retrieval of foreign bodies, dura repair was performed. Patient was discharged from the hospital with partial recovery. Operative decompression of the neural elements in case of spinal canal compromise is the treatment of choice. Indication for surgical intervention in existing cerebrospinal fluid fistula includes closure of the dura and reducing neural elements compression and lowering the risk of infectious complications by removing bone or foreign body fragments. Patients with Brown-Séquard syndrome have good prognosis for functional recovery.

## Introduction

Injuries in children are one of the most frequent causes of high morbidity and mortality, and they present a challenge to the treating physician. Fortunately, spinal trauma in pediatric patient is relatively rare. Our knowledge about the differences between pediatric and adult spinal column and spinal cord injuries remains still incomplete; therefore, each case provides us with valuable new information. All types of spinal cord injuries are present in children population as in adults. Brown-Séquard syndrome, first described in 1846 by Charles-Edouard Brown-Séquard [1], is a rare form of incomplete spinal cord injury consisting of ipsilateral upper motor neuron paralysis (hemiplegia) and loss of proprioception with contralateral pain and temperature sensation deficits resulting from hemisection or lateral injury to the spinal cord. Typically, it occurs in young males aged 15–50 years old after penetrating trauma [2]. There are very few cases of Brown-Séquard syndrome after a penetrating trauma to the spine in children reported in literature. In this population, motor vehicle accidents or other blunt traumas are the most common causes of spinal injury. We present a case of Brown-Séquard syndrome after a stab wound to the thoracic region from broken glass window.

## Case report

A 11-year-old girl, with slight degree of mental retardation, was admitted to our Pediatric Trauma Emergency Department after she had suffered a penetrating back injury. She was running in her house when she suddenly bumped to the glass door. She has not lost consciousness.

**Fig. 1:**
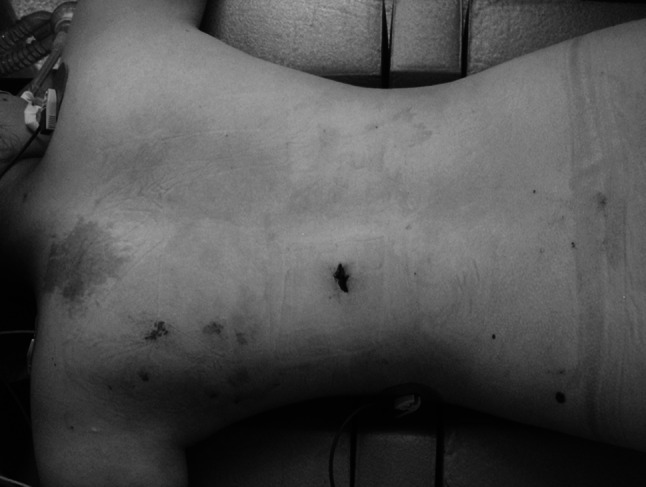
Stab wound in the *left* thoracic region of the back

On examination at admission, she was in logical contact. She was 15 points in Glasgow Coma Scale. The vital signs were heart rate 84 beats/min, blood pressure 100/70 mmHg, respiratory rate 20 breaths/min and temperature 36.6 °C. Physical examination revealed a linear 1.5-cm-long stab wound in the low left paraspinal thoracic region (Fig. 1).

There was no foreign body seen over the skin surface. Bleeding was minimal, but cerebrospinal fluid leakage from the wound was observed. No other injuries were diagnosed. Neurological examination demonstrated left-side lower extremity paresis and moderate spastic paralysis on the other lower extremity. Bilateral positive Babinski sign was observed. An exact sensory examination was not possible because of slight mental retardation, but loss of temperature sensation contralateral to and below the lesion was noticed. The examination of the pain was difficult because the patient was in pain shock, but it was diminished on the side opposite to the damage. Rectal sphincter muscle tone was normal. The urogenital examination was normal. There were no abnormalities in laboratory tests.

**Fig. 2:**
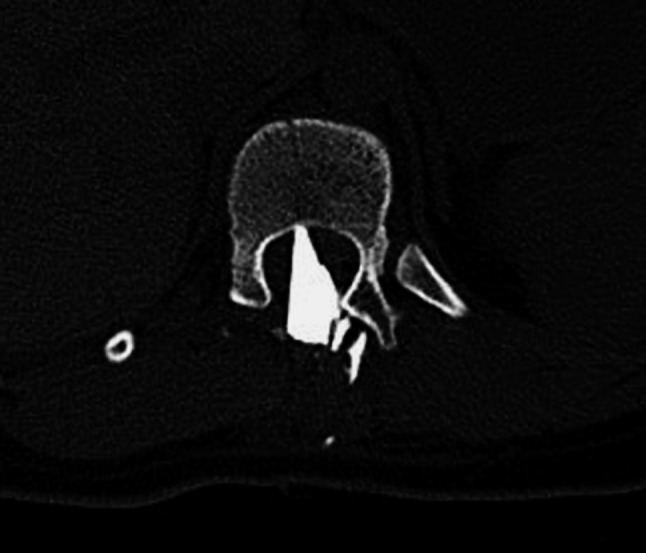
CT scan Th11–Th12. A piece of glass window penetrating to the spinal canal

Multislice spiral computed tomography (MSCT) demonstrated a triangular foreign body inside the spinal canal (26 mm × 11 mm × 6 mm) at the level of the Th_11_–Th_12_ and several small fragments of the glass in the paraspinal muscles on the left side (Fig. 2). Steroids were not used, and standard perioperative antibiotic prophylactics were administered. The patient was immediately taken to the operating theatre. The wound was explored. A Th_11_–L_2_ laminectomy was performed. Intraoperatively the 2-cm-long laceration of the dura was diagnosed, and the spinal cord was also injured.

**Fig. 3:**
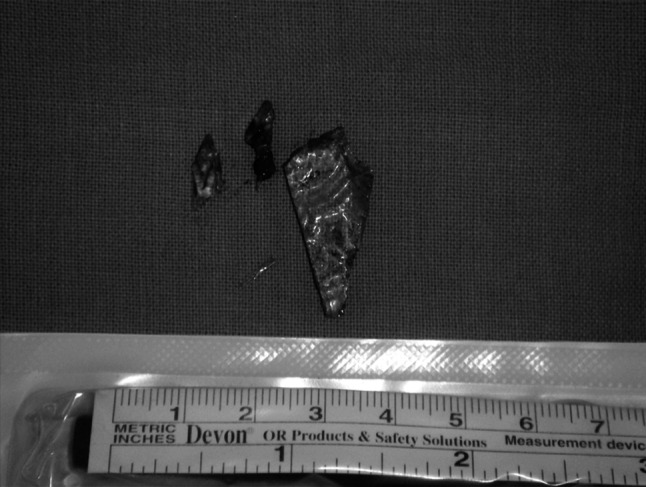
Removed pieces of the glass window

After the foreign bodies retrieval (Fig. 3), dura laceration repair was performed. There were no signs of the cerebrospinal leakage after the dura closure. Postoperative course was uncomplicated. The girl stayed in Pediatric Surgery Department for 16 days. The rehabilitation started in the 3rd day after the surgery.

At the discharge date, she was partially recovered. The wound healed uneventfully. Muscle strength was rated according to 0–5 Lovett score [3] as follows:

- 1/5: muscle flicker, but no movement
- 2/5: movement possible, but not against gravity
- 3/5: movement possible against gravity, but not against resistance by the examiner
- 4/5: movement possible against some resistance by the examiner
- 5/5: normal muscle strength.

Partial paresis in the left lower limb (3/5 in the Lovett score) and slight paresis of the right lower limb (4/5 in the Lovett score) were present. Positive bilateral Babinski sign was still present. The girl was ambulating with assistance of the walker.

She was discharged from the Pediatric Surgery Department and referred to the Rehabilitation Ward.

## Discussion

Brown-Séquard syndrome is a result of disruption of the descending lateral corticospinal tracts and the ascending lateral spinothalamic tracts, which cross within one or two levels on the dorsal root entrance [4]. Clinically, they result in an ipsilateral to injury loss of position and vibration sensation as well as paralysis and hyperesthesias and contralateral loss of pain and temperature sensation. Light touch sensation is typically not affected [2].

Spinal injury is an uncommon source of morbidity in children. The most common cause of spine injury is motor vehicle accident, falls and sports-related accidents [5, 6]. Far less common is penetrating injury to the spine, with the stab wounds and gunshot traumas being the most common reasons in adults. Only about 3 % of traumatic spinal cord injury ends up with Brown-Séquard syndrome; however, a pure form of this syndrome occurs rarely and therefore the term ‘Brown-Séquard-plus’ syndrome is used when additional neurological findings are present [7]. Several cases of such Brown-Séquard-plus syndrome have been reported in adult literature, most of them caused by penetrating trauma and rarely by blunt trauma [8]. Reports concerning children population are very infrequent.

Treatment for this disease should be the same as for other acute spinal cord injuries. Indication for surgical intervention in existing cerebrospinal fluid fistula includes closure of the dura and reducing neural elements compression and lowering the risk of infectious complications by removing bone or foreign body fragments or hematoma [9, 10]. However, severe neurological deficits remaining unchanged from the time of injury rarely improve after decompressive surgery.

Randomized prospective trials in adults (NASCIS 3) suggest that patients with motor impairment may benefit from high-dose steroids administration within 8 h of an injury, but their role is controversial [11]. There are no randomized, prospective trials concerning steroid use in spinal cord injury in children population. Steroid use is even more controversial in penetrating injury due to increased risk of infections leading to prolonged hospital stay [12]; nevertheless, some authors advocate treatment with methylprednisolone succinate in scheme as in adult.

In many cases of spinal cord injuries, different types of loss of motor, sensory and autonomic functions below the level of lesion are observed. Patients with Brown-Séquard syndrome have relative good prognosis for functional recovery [13].
